# Harnessing the Potential of Forage Legumes, Alfalfa, Soybean, and Cowpea for Sustainable Agriculture and Global Food Security

**DOI:** 10.3389/fpls.2018.01314

**Published:** 2018-09-19

**Authors:** Krishnanand P. Kulkarni, Rupesh Tayade, Sovetgul Asekova, Jong Tae Song, J. Grover Shannon, Jeong-Dong Lee

**Affiliations:** ^1^School of Applied Biosciences, Kyungpook National University, Daegu, South Korea; ^2^Department of Southern Area Crop Science, National Institute of Crop Science, Rural Development Administration, Miryang, South Korea; ^3^National Center for Soybean Biotechnology and Division of Plant Sciences, University of Missouri, Columbia, MO, United States

**Keywords:** forage legumes, forage yield, forage quality, alfalfa, soybean, cowpea, quantitative trait loci, genetic manipulation

## Abstract

Substantial improvements in access to food and increased purchasing power are driving many people toward consuming nutrition-rich foods causing an unprecedented demand for protein food worldwide, which is expected to rise further. Forage legumes form an important source of feed for livestock and have potential to provide a sustainable solution for food and protein security. Currently, alfalfa is a commercially grown source of forage and feed in many countries. However, soybean and cowpea also have the potential to provide quality forage and fodder for animal use. The cultivation of forage legumes is under threat from changing climatic conditions, indicating the need for breeding cultivars that can sustain and acclimatize to the negative effects of climate change. Recent progress in genetic and genomic tools have facilitated the identification of quantitative trait loci and genes/alleles that can aid in developing forage cultivars through genomics-assisted breeding. Furthermore, transgenic technology can be utilized to manipulate the genetic makeup of plants to improve forage digestibility for better animal performance. In this article, we assess the genetic potential of three important legume crops, alfalfa, soybean, and cowpea in supplying quality fodder and feed for livestock. In addition, we examine the impact of climate change on forage quality and discuss efforts made in enhancing the adaptation of the plant to the abiotic stress conditions. Subsequently, we suggest the application of integrative approaches to achieve adequate forage production amid the unpredictable climatic conditions.

## Introduction

Food security faces threats from climate change and weather variability and needs immediate consideration, especially in geographic regions where agriculture is highly dependent on rainfall (Khanal and Mishra, 2017). Recent changes in climate are of global concern for availability of sufficient food not only for humans but also for animals (Wheeler and Von Braun, 2013). Livestock contributes ∼40% of the global value in agricultural production and provides nutrition and food security to humans (Smith et al., 2014). It is the largest user of land resources worldwide, with almost 80% of the global arable land dedicated to production of animal feed (FAO, 2017b). Because of substantial improvements in access to food in many developing countries and increased purchasing power allowing many people to consume protein-rich and nutritious food (FAO, 2017c), per capita meat consumption is predicted to increase markedly. Worldwide total livestock production has shown a > 200% increase in the last 54 years (FAOSTAT, 2017). As the global human population is expected to surpass 9 billion by 2050 (UN estimates), it is predicted that there would be an unprecedented rise in demand for food of animal origin (National Research Council, 2015). In such a scenario, striking a balance between cultivation of grain/field crops for humans and forage crops for animal feed could be challenging.

Due to their high dry matter production, cereal crops have been used over many centuries as a feed for ruminant animals. However, they are poor in protein content, and hence often considered low-quality forage sources. On the other hand, forage legume crops provide high-quality fodder and feed for livestock. When cereal crops are grown together with legume crops, which can improve yield as well as protein content and other quality parameters (Zhang J. et al., 2015), forage with adequate nutritive value can be produced. The legume crops have the ability to fix atmospheric nitrogen (N) for plant use owing to the symbiotic nature of the plant-*Rhizobium* relationship (Collins et al., 1986). The estimated amounts of N fixed from atmospheric N in legume/grass pastures in the world range from 13 to 682 kg N ha^-1^ year^-1^ (Ledgard and Steele, 1992). With the increased interest in low-input sustainable agriculture in the world, possibly due to the environmental problems associated with the application of high doses of N fertilizers for yield improvements, increased cultivation of legume crops may offer potential benefits.

As many as 60 different legume crops have been cultivated as sources of forage and feed for animals. Currently, alfalfa (*Medicago sativa* L.), a perennial legume crop, is the most frequent and commercially grown source of forage and feed in many countries. However, alfalfa may not be the priority crop across geographic regions, and other legume crops providing quality forage may be preferred. For instance, in sub-Saharan Africa (particularly in West Africa) and India, cowpea [*Vigna unguiculata* (L.) Walp.] has been an integral part of traditional cropping systems where grains are used as food and haulms are fed to livestock as a nutritious fodder (Singh et al., 2003). Other legume crops, such as Austrian winter pea (*Pisum sativum* subsp. Arvense), white clover (*Trifolium repens* L.), and sweet clover (*Melilotus albus* L.) have been favored in different regions in the United States. Hence, given the inevitability of climate change, it is crucial to assess the forage potential of different legume crops in terms of forage quality parameters, digestibility and animal performance, and feasibility of sustainable growth and consistent yields. In this review, we assess the effects of climate change on forage quality, the efforts made in identifying the genetic basis of forage quality and yield traits, and genetic manipulation to improve forage digestibility in three important legume crops, alfalfa, soybean [*Glycine max* (L.) Merr.], and cowpea.

Alfalfa, also called lucerne, is an autotetraploid, perennial legume crop grown worldwide for its high-quality forage and nutritional value (Li and Brummer, 2012). Most cultivated alfalfa varieties are derived from either subsp. *sativa* or subsp. × *varia*, with some direct use of subsp. *falcata per se* or in hybrids with the other subspecies (McCoy and Bingham, 1988; Small, 2010). Alfalfa can be genetically hybridized to develop several other members of the genus, mainly perennial species. The perennial alfalfa provides maximum yield during the second year of cultivation (FAO, 2017a). In geographic regions with mild winters, alfalfa can be grown continuously for 3–4 years; however, in climates with cold winters, it can be grown for 6–9 years, with a dormant period in winter (FAO, 2017a). Forage quality and yield, lodging resistance, and early spring vigor are considered the most important traits in alfalfa improvement (McCord et al., 2014). Besides, alfalfa plants have the ability to fix up to 350 kg N ha^-1^ (Carlsson and Huss-Danell, 2003). Due to these benefits, alfalfa is grown throughout the dry tropical and temperate regions of the world (Monteros et al., 2003).

Soybean, on the other hand, is an annual legume domesticated primarily for its high seed protein and oil content (Patil et al., 2017). Soybean has a long history of being grown as a forage crop (Blount et al., 2009). In North East Asia, fresh wild soybean, dried wild soybean, and after threshing mature soybean pods are used as feed source for animal. It is noteworthy that the major usage of soybean following introduction into the United States in the mid-1800s was as a forage crop (Probst and Judd, 1973). Soybean acreage for grain in the United States first exceeded the acreage for forage in 1941 due to the growing demand for soybean oil and meal (Nielsen, 2011). However, interest has grown in utilizing soybean as a forage crop in the last two decades. As a forage crop, it has several advantages, such as high protein content at the seed filling stage, a wide range of growth stages suitable for harvest, efficient cover to reduce soil erosion, and wide adaptability to different climatic zones (Asekova et al., 2014, 2016b). Soybean is estimated to fix on average 79 kg N ha^-1^ (Salvagiotti et al., 2008). Soybean forage harvested at growth stages from R5 (seed development) to R7 (beginning maturity) (Fehr et al., 1971) has been considered highly suitable for animal feed, as it has the best combination of high protein, low fiber content, and greater digestible energy (reviewed in Asekova et al., 2014).

Cowpea is another important annual legume crop, which is a key food resource for millions of people living in tropical and subtropical regions (Tshovhote et al., 2003). Although cowpea has its origin in sub-Saharan Africa, it is now cultivated in over 100 countries (Singh, 2014). It is an equally important and nutritious fodder crop for livestock and is often recommended as an alternative source of protein and energy for animals during the winter and dry seasons (Singh et al., 2003). It has the potential to be an important forage and fodder crop for the future, as it can grow and adapt to regions with sandy and relatively infertile soils and low rainfall (Singh and Tarawali, 1997). Utilization of cowpea as fodder is most widespread in Asia, especially in India, where green cowpea plants are either used for grazing or cut and used in combination with dry cereals for feeding animals. In particular, the use of cowpea haulms as fodder is highly attractive in mixed cropping systems where both the grain and fodder can be obtained from the same crop (Tarawali et al., 1997). In West Africa, after harvesting mature cowpea pods, the haulms are cut while still green and rolled into small bundles for storing for later use as a feed source (Singh and Tarawali, 1997). Cowpea is estimated to fix atmospheric N in total between 70 and 350 kg ha^-1^, contributing 40–80 kg N ha^-1^ (Quin, 1997) to the soil to improve its fertility. Nutritionally, the value of cowpea green pods and green leaves is recognized due to their high protein and low-fat contents (Gonçalves et al., 2016).

## Impact of Climate Change on Legume Forage Quality

Climate change not only dramatically alters forage nutritive value and yield but also affects livestock health (Craine et al., 2010; Baumgard et al., 2012). The adverse climatic conditions may expose forage crops to various abiotic stresses affecting the forage quality and yield. Several studies have reviewed the influence of environmental factors, such as light, temperature, drought, and soil nutrients on chemical composition and digestibility of forage crops grown in different areas of the world. Hopkins and Del Prado (2007) investigated implications of climate change associated with greenhouse gas emissions, increased temperature, and elevated CO_2_ levels for grassland and recommended the need for several future agricultural adaptations to ensure adequate forage availability for feed production. Although elevated CO_2_ levels increase photosynthesis and yield, the photosynthetic response of forage plants to CO_2_ generally decreases under long-term exposure, and plants tend to acclimate to elevated CO_2_ concentrations (Sage et al., 1989), which is referred to as downregulation (Saralabai et al., 1997). Such photosynthetic downregulation affects forage yields, especially in perennial forage crops such as alfalfa (**Table 1**). While increases in CO_2_ concentration have only small effects on forage solubility and fiber (cellulose and lignin) components, they strongly affect crude protein (CP) concentrations and forage digestibility (Milchunas et al., 2005). Dumont et al. (2015) performed a meta-analysis of 75 studies to identify the effects of climate change on the quality of forage in grasslands. Their analysis indicated that elevated CO_2_ levels increased total non-structural carbohydrates by 25% and decreased N content by 8% in forage grass tissues.

**Table 1:** Effect of different climatic factors on forage quality and yield in alfalfa.

Environmental factor	Geographical location	Soil type	Traits	Significant findings	Reference
CO_2_ + high temperature	Pamplona, Spain		Forage digestibility, FQ, FY	Reduced digestibility and CP; enhanced fiber content	Sanz-Saez et al., 2012
CO_2_	Barcelona, Spain		FQ, stem carbohydrates	Leaf, stem, and root biomass, hemicellulose and lignin content affected in non-mycorrhizal alfalfa plants	Baslam et al., 2012
Drought	Ames, Iowa, United States	Nicollet loam top soil (fine-loamy, mixed)	FQ	Increase in leaf to stem ratio, stem IVDDM, and CP; reduction in maturity	Halim et al., 1989
Drought	Becker, Minnesota, United States	Hubbard loamy sand	FY, ADF, NDF, and ADL	Reduction in yield potential in drought conditions	Peterson et al., 1992
Drought	Ames, Iowa, United States		Cell wall composition, structural polysaccharide degradability	4.8 and 2.9% increases in cell wall and Klason-lignin concentrations with increase in maturity; no influence on cell-wall degradability	Deetz et al., 1996
Drought	Las Cruces, New Mexico	Glendale clay loam (fine, silty, and mixed)	WUE, DMY, maturity, and leaf/stem ratio	Increase in DMY; early maturity; reduced leaf/stem ratio.	Ray et al., 1999
Drought	Jiroft, Iran		FY, FQ	Decrease in FY; increase in leaf/stem ratio	Afsharmanesh, 2009
Drought			Molecular, biochemical, and physiological responses	Delayed leaf senescence, greater root growth, and greater accumulation of osmolytes	Kang et al., 2011
Elevated CO_2_, temperature, and drought	CSIC, Salamanca, Spain		Photosynthesis during vegetative normal growth	Photosynthetic acclimation; no effect in re-growth	Erice et al., 2006
Inorganic N supply			FQ	No difference in forage digestibility, NDF, ADF, and lignin; increase in N concentration of alfalfa plants	Cherney et al., 1994
Soil hydrologic conditions	New York, United States	Sandy clay loam soil	FY, FQ	Reductions in the yield, fiber, and lignin; increase in CP	Buscaglia et al., 1994
Soil moisture and ambient temperature	Minnesota, United States	Fertile sandy-loam soil	FY, FQ	Reduced dry matter and lower digestibility; higher ADF/ADL at high temperatures	Vough and Marten, 1971
Soil moisture deficit	Becker, Minnesota, United States	Hubbard loamy sand	FQ, forage digestibility, and protein fractionation	Increase in ADF; reduced ADL content; increased forage digestibility	Seguin et al., 2002
Soil water deficits	Becker, Minnesota, United States	Hubbard loamy sand (sandy, mixed)	Growth, FY, FQ	Decrease in DMY; increase in leaf/stem ratio	Carter and Sheaffer, 1983
Variable irrigation	Sudan	Clay soil (40%) with high water-holding capacity	Growth, yield, WUE	Reduction in stem height, biomass yield and WUE	Saeed and El-Nadi, 1997
Water supply, temperature, CO_2_	CSIC, Salamanca, Spain		Photosynthesis	Enhanced photosynthetic rate	Aranjuelo et al., 2005


*ADF, acid detergent fiber; ADL, acid detergent lignin; CF, crude fat; CP, crude protein; DMY, dry matter yield; FQ, forage quality; FY, forage yield; IVDMD, *in vitro* dry matter digestibility; N, nitrogen; NDF, neutral detergent fiber; NDFD, neutral detergent fiber digestibility; WUE, water use efficiency.*

Apart from CO_2,_ the increases in ground-level concentrations of ozone (O_3_) are also expected to contribute to global warming. O_3_ acts as a shield in the stratosphere by providing protection from lethal shortwave solar ultraviolet radiation but is an air pollutant and greenhouse gas in the troposphere. It poses a critical threat and is a challenging problem for world food security and plant habitats (Ashmore, 2005). Plant exposure to elevated concentrations of ground-level O_3_ suppresses photosynthesis, accelerates senescence, reduces plant growth, and causes inferior yields (Booker et al., 2009). The yield and quality of forage crops may also be detrimentally affected by O_3_, which could have negative consequences for animal production. The residual effects of O_3_ caused a reduction in yield in the subsequent cutting of alfalfa (Hoffman et al., 1975). The influence of O_3_ on productivity and nutritive quality has been studied in other forage crops such as ryegrass (*Lolium perenne* L.), white clover and subterranean clover (*Trifolium subterraneum* L.) (Blum et al., 1983; Nussbaum et al., 1995; Sanz et al., 2005; González-Fernández et al., 2008). These studies recorded adverse effects of O_3_ on forage-associated traits, such as impairment of the aerial/subterranean biomass ratio, increases in acid detergent fiber (ADF) concentrations, reduction in the nutritive quality of aerial biomass, acceleration of senescence, and reduced regrowth and total forage production. From these studies, it can be inferred that efforts to develop international control policies to reduce exposure to ozone will be needed in the near future (Fuhrer et al., 1997).

It is well documented that abiotic stresses cause different kinds of phenotypic, biochemical, and molecular alterations that severely impair plant growth and development, productivity, and yield (Bita and Gerats, 2013). Plants are regularly subjected to such stresses at one or more stages during growth, which may affect the quality and nutritive value in forage legume crops. Drought, along with rising temperature, causes photosynthetic acclimatization in alfalfa plants (Erice et al., 2006). Soil water deficit has been shown to reduce not only yield (Peterson et al., 1992; Saeed and El-Nadi, 1997; Afsharmanesh, 2009), but also forage quality in alfalfa (Buscaglia et al., 1994; Cherney et al., 1994; Seguin et al., 2002; Baslam et al., 2012; **Table 1**). Kang et al. (2011) analyzed molecular, biochemical, and physiological responses of alfalfa plants exposed to drought conditions, and observed several effects, such as delayed leaf senescence, greater root growth, and greater accumulation of osmolytes, including raffinose and galactinol, and flavonoid antioxidants in roots and/or shoots. Such responses may be at the expense of other characteristics that are beyond estimation. Similar to drought, heat stress adversely affects the vegetative stages of plant growth, which may cause large yield losses in forage crops. High temperatures are also reported to reduce leaf/stem ratios and forage digestibility (Buxton, 1996). Similar responses could be detected when plants are subjected to salt and drought stresses, although responses in terms of morphological and physiological changes may differ.

The advancements in the genetic and genomics-assisted breeding and biotechnological approaches offer exceptional prospects to enhance the adaptation of the plants to climatic conditions such as drought and salinity stresses (recently reviewed by Kole et al., 2015; Abberton et al., 2016; Batley and Edwards, 2016; Dhankher and Foyer, 2018). Breeding efforts in forage legume crops such as alfalfa are difficult due to relatively high levels of genetic variation and environmental interactions (Annicchiarico et al., 2015). Hence, the most of the trait improvements in alfalfa have been made through biotechnological approaches. The transgenic expression of genes such as *Alfin1* (encoding a putative transcription factor; Winicov and Bastola, 1999), *WXP1* (encoding an ethylene-responsive element-binding transcription factor; Jiang et al., 2009), *GmDREB1* (encoding a dehydration responsive element-binding protein; Jin et al., 2010), *BADH* (encoding for betaine aldehyde dehydrogenase; Liu et al., 2011), *GsZFP1* (encoding a Cys2/His2-type zinc-finger protein; Tang et al., 2013) and *GsWRKY20* (encoding a WRKY-type transcription factor; Tang et al., 2014), *EsMcsu1* (encoding a molybdenum cofactor sulfurase from *Eutrema salsugineum*; Zhou et al., 2015), Arabidopsis *ABF3* (An *ABSCISIC ACID-RESPONSIVE ELEMENT-BINDING FACTOR 3* gene encoding a bZIP transcription factor; Wang et al., 2016b), and Arabidopsis *EDT1* (*Arabidopsis Enhanced Drought Tolerance 1*; Zheng et al., 2017) has been found to improve the growth of alfalfa transgenic plants in different abiotic stress conditions. Similarly, *MicroRNA156* has been found to improve drought stress tolerance in alfalfa (Arshad et al., 2017) and heat stress (Matthews et al., 2018) by silencing SPL13. Conversely, biotechnological improvement of soybeans has been reported for genes such as a *L*-Δ*1-Pyrroline-5-carboxylate reductase* (De Ronde et al., 2000, 2004) conferring improved drought tolerance, and the *Panax ginseng PgTIP1* gene conferring enhanced salt and drought tolerance (An et al., 2018). The recent progress in functional genomics and in understanding the mechanisms of abiotic stress tolerance in model crop like Arabidopsis (*A. thaliana*) and rice (*Oryza sativa*) has opened a way for genetic manipulation of soybean plants with abiotic stress tolerance (Thao and Tran, 2012). The success of developing transgenic plants depends on the availability of transformation and regeneration protocols, which are yet to be fully established for soybean and cowpea. The availability of a number of genetic and genomic resources in both the crops is expected to accelerate genetic engineering and molecular breeding efforts for breeding climate-resilient varieties. The abiotic stresses, including drought and salinity, are complex traits and require a thorough assessment of physiological response variability among the available germplasm in order to identify and focus on the traits that are crucial for high yields under the changing climate conditions. Further understanding of the genetics of these traits as well as forage-related traits would be helpful for the breeders to develop improved varieties that combine abiotic stress tolerance with forage traits using marker-assisted selection.

## Genetic Dissection of Forage Traits in Legumes

Recent progress in next-generation sequencing technologies has facilitated discovery of a large number of single nucleotide polymorphism (SNP) and simple sequence repeat (SSR) markers widely distributed across the genomes of agronomically important crops (Kulkarni et al., 2012). Further, the advent of genotyping technologies such as array-based chip platforms has expedited genotyping of a large number of samples to generate large datasets in a short period. Such data can be useful for developing high-density bin maps and high-resolution linkage maps as well as for genome-wide association analyses to identify chromosomal loci governing complex agronomic traits. Approaches such as quantitative trait locus (QTL) mapping and genome-wide association study (GWAS) help in identifying marker-trait associations that can guide marker-assisted selection (MAS) and/or genomic selection (GS) in breeding programmes (Zhao et al., 2011; McCord et al., 2014). Despite these advancements, and with the exception of alfalfa, the progress in genetic dissection of forage traits such as shoot fresh and dry weight, yield, and forage quality parameters in other legume crops has gained little pace. Of the three crops we considered in this review, alfalfa is commercially grown for forage production and is perennial in nature, a trait that suits its use for forage, feed, and other applications. However, soybean and cowpea are not grown as forage crops at a commercial scale similar to that of alfalfa. Here, we reviewed efforts made in the past few decades to reveal the genetic basis of forage quality and yield traits in alfalfa as well as in soybean and cowpea. Such information can be useful for devising further strategies to improve forage quality and yield in these three important crops.

### Genetic Mapping of Forage Traits in Alfalfa

The genetic dissection in tetraploid alfalfa was complicated due to the tetrasomic inheritance and the difficulties in analyzing polyploid linkage relationships (Sakiroglu et al., 2012). Thus, the earlier genetic linkage maps were developed in diploid (2n = 2× = 16) species of alfalfa (Brummer et al., 1993; Echt et al., 1993; Kiss et al., 1993; Diwan et al., 2000), using molecular markers such as restriction fragment length polymorphism, random amplified polymorphic DNA, and SSR markers (Brouwer et al., 2000; Julier et al., 2003; Sledge et al., 2005). The advances in the marker development, as well as linkage analysis tools, such as the development of TetraploidMap (Hackett and Luo, 2003) greatly facilitated genetic mapping in tetraploid alfalfa. Several QTLs for important traits such as winter hardiness, fall growth and freezing injury (Brouwer et al., 2000), biomass production (Robins et al., 2007a), forage yield (Robins et al., 2007b; McCord et al., 2014), plant height and regrowth (Robins et al., 2007b), persistence tolerance (Robins et al., 2008) and, lodging resistance and spring vigor traits (McCord et al., 2014) have been identified in tetraploid alfalfa (**Table 2**). In addition, Ray et al. (2015) identified small-effect QTLs controlling biomass yield under drought in tetraploid alfalfa (**Table 2**).

**Table 2:** Details of genetic dissection studies for forage quality and yield-related traits in alfalfa and soybean.

Crop	Traits analyzed	Plant materials	No. of env.	Mapping approach (marker system)	No. of markers	Chr.	PVE (%)	No. of QTL/SNP	Reference
Alfalfa	WH, fall growth, and freezing injury	101 plants each from two BC populations (B17 × P13)		Single marker analysis (RFLP)	82		6.3–52.2	1–6	Brouwer et al., 2000
	Biomass production	200 F_1_ plants (WISFAL-6 × ABI-408)	3	Single marker analysis (RFLP, SSR)				41	Robins et al., 2007a
	FY, PH, and forage regrowth	200 F_1_ plants (WISFAL-6 × ABI-408)	3	Linkage mapping		1–8	11–44	86	Robins et al., 2007b
	Persistence tolerance	200 F_1_ plants (WISFAL-6 × ABI-408)	2	Linkage mapping		1–3, 7	8–13	16	Robins et al., 2008
	FQ and stem histology	Four RIL populations of *M. truncatula* sharing common parents		Linkage mapping		1, 3, 7, 8		86	Espinoza and Julier, 2013
	FY, lodging resistance, spring vigor	BC_1:3_ (128 plants from DW000577 × NL002724)	4	Linkage mapping (AFLP, SRAP, and SSR)	236	3–8	9.4–27.9	6	McCord et al., 2014
	Forage biomass productivity under drought	Two BC_1_ populations (comprising 133 and 120 plants) from the WF1 × CH28		Linkage mapping (EST-SSR, SSR, and SNP)	539	1–3, 5, 6, 8	2.8–8.1		Ray et al., 2015
	BY, ADF, ADL, NDF, and stem composition	190 tetraploid plants selected from a strain cross between 3 varieties	2	Association mapping (SSR)	71	1, 3–8	2–6	17	Li et al., 2011
	Lignin biosynthesis genes, ADF, NDF, ADL, TNC, PH, and BY	374 individual genotypes from 120 accessions	2	Association mapping (SSR) and candidate gene resequencing	89	4–6		7	Sakiroglu et al., 2012
	BY under drought (greenhouse)	198 cultivars and landraces from USDA-ARS NPGS alfalfa collection	1	Association mapping (SNP from GBS)		1–7		19	Zhang J. et al., 2015
	NDF, ADF, NDFD, and leaf/stem ratio	154 genotypes from the strain cross between 3 cultivars	3	Association mapping (SNP)	11450	1, 2, 4, 5, 7, 8	10–20.2	83	Biazzi et al., 2017
	FQ traits	336 cultivated tetraploid alfalfa genotypes	4	Association mapping (SSR)	85	1–5, 7	2.48–9.66	124	Wang et al., 2016a
	CP, mineral concentration	336 alfalfa genotypes		Association mapping (SSR)	85		2.1–4.09	2–8	Jia et al., 2017
	BY (drought in field conditions)	200 alfalfa accessions selected from the USDA-ARS NPGS alfalfa collection	2	Association mapping (SNP from GBS)		1–8	7–32	28	Yu, 2017
	SS, DW, LCC, PH, and SC	198 accessions selected from the USDA-ARS NPGS alfalfa collection		Association mapping (SNP from GBS)		1, 3, 5, 7	8–38	42	Liu and Yu, 2017
	FY, nutritive value	362 plants (from 120 diploid accessions of the *M. sativa*–falcata complex)	2	Association mapping (SNP)	15154			65	Sakiroglu and Brummer, 2017
Soybean	SFW, SDW, and SFW/SDW	94 RILs (F_5_) from “Essex × Forrest”	1	Linkage mapping (SSR, RFLP, RAPD, and AFLP)	237	2, 3, 5, 6, 8–10, 12	12–34	10	Brensha et al., 2012
	SFW	188 RILs (F_5:8_) from an interspecific cross of PI 483463 × Hutcheson	4	Linkage mapping (SSR, SNP)	551	6, 15, 19	6.6–21.3	3	Asekova et al., 2016b
	CP, CF, NDF, and ADF	188 RILs (F_5:8_) from an interspecific cross of PI 483463 × Hutcheson	4	Linkage mapping (SSR, SNP)	551	7, 11, 12, 14, 15, 19	5.8–41.7	16	Asekova et al., 2016a


*ADF, acid detergent fiber; ADL, acid detergent lignin; AFLP, amplified fragment length polymorphism; BY, biomass yield; CF, crude fat; Chr., chromosome; CP, crude protein; DMY, dry matter yield; DW, dry weight; Env, environment; EST, expressed sequence tag; FQ, forage quality; FY, forage yield; GBS, genotyping by sequencing; GWAS, genome wide association mapping; IVDMD, *in vitro* dry matter digestibility; LCC, leaf chlorophyll content; NDF, neutral detergent fiber; NDFD, neutral detergent fiber digestibility; PH, plant height; PVE, phenotypic variation; QTL, quantitative trait loci; SFW, shoot fresh weight; SDW, shoot dry weight; SNP, single nucleotide polymorphism; SS, salt stress; SC, stomatal conductance; SRAP, Sequence-related amplified polymorphism; SSR, simple sequence repeat; RAPD, random amplified length polymorphism; RFLP, restriction fragment length polymorphism; RIL, recombinant inbred line; WH, winter hardiness; WUE, water use efficiency.*

*Medicago truncatula* is a model plant species and can be used to map and clone genes in legume crops. The availability of the genome sequence for *M. truncatula*, which has a high syntenic relationship with cultivated alfalfa, may provide comprehensive information on genetic inheritance and functional genomics of agronomically important traits. In forage legumes, aerial morphogenesis is an important trait, which determines plant and seed biomass as well as forage quality (Julier et al., 2007). Using recombinant inbred line (RIL) populations derived from different accessions of *M. truncatula*, several major QTLs affecting aerial morphogenetic traits have been identified (Julier et al., 2007; Espinoza et al., 2012). In addition, QTLs for different forage quality traits and stem histology (Espinoza and Julier, 2013) using *M. truncatula* have been detected. Similarly, Pierre et al. (2008) identified six candidate genes from a major QTL consistent across the three RIL populations segregating for flowering date, the trait important for adaptation to the environment. The above-mentioned QTLs can be further evaluated for their additive effects under different environments and explored to identify candidate genes, which can help either in trait improvement by introgression into the desired cultivars or in genetic manipulation studies to identify and validate gene function and phenotype effects.

Association mapping has been seen as a promising methodology for trait mapping due to the availability of a large number of identified SSR/SNP markers and high throughput genotyping platforms (Gupta et al., 2014). GWAS enables detection of marker-trait associations in unstructured populations or wild germplasm collections by taking advantage of linkage disequilibrium (LD) existing between a marker and the true causative polymorphism of the trait phenotype (Breseghello and Sorrells, 2006). Because natural populations exhibit greater genetic diversity and represent historic recombination over many generations, a large number of QTLs can be detected with precise chromosomal locations. In addition, association mapping provides higher mapping resolution, and detects a greater number of alleles than other methods (Zhu et al., 2008). Therefore, this approach has increasingly been favored in identifying chromosomal loci controlling a particular phenotype expression under certain environmental conditions. In the last few years, several association mapping studies have been conducted using diploid or tetraploid alfalfa breeding populations to identify marker-trait associations for biomass yield and stem composition (Li et al., 2011; Sakiroglu et al., 2012), fiber-related traits and digestibility (Wang et al., 2016a), and CP concentrations (Jia et al., 2017) (**Table 2**).

Genotyping-by-sequencing (GBS), a cost-effective molecular marker genotyping method, has been widely preferred in GWAS analysis to investigate marker-trait associations in different plant species, including alfalfa. Zhang T. et al. (2015) used this approach to identify 19 loci associated with drought resistance traits in a heterozygous autotetraploid alfalfa population. Many of these loci were found to overlap with the reported QTLs associated with biomass yield under drought. Similar marker-trait associations have been identified for forage yield and nutritive value (Sakiroglu and Brummer, 2017), forage quality traits (Biazzi et al., 2017), plant growth and forage production (Liu and Yu, 2017), and biomass yield under water deficit conditions (Yu, 2017; **Table 2**). Thus, genetic dissection of forage-related traits using advanced approaches could be useful in identifying loci that can be exploited through marker-assisted breeding to develop drought-resistant alfalfa cultivars with improved nutritive value.

### Genetic Mapping of Forage Traits in Soybean

Compared to alfalfa, little attention has been given to soybean as a forage crop, and hence there are few studies of genetic dissection of forage traits. Brensha et al. (2012) utilized a RIL (F_5_) population of 94 plants derived from the Essex × Forrest cross to identify markers associated with shoot fresh weight (SFW), shoot dry weight (SDW), and SFW/SDW ratio (**Table 2**). Using composite interval mapping, they identified four QTLs for SFW, three QTLs for SDW, and three QTLs for SFW/SDW, explaining 12–34% of the total phenotypic variation. The study utilized a cross of two cultivated soybeans differing in many traits, including root and shoot traits. However, wild soybeans (*Glycine soja*) have also been shown to possess alleles that can contribute positively to different agronomically important traits in cultivated soybean (Ha et al., 2013, 2014; Kulkarni et al., 2016, 2017a; Patil et al., 2016). Lee et al. (2014) observed that some of the RILs from an interspecific cross of PI 483463 (a wild soybean accession, with thinner main stems and branches and good forage yield) and “Hutcheson” had higher feeding value than cultivated soybeans, which suggested that the wild soybean PI 483463 may have favorable alleles that could be utilized to improve forage traits. Therefore, these RILs were utilized for genetic dissection studies, in which 3 QTLs for SFW exhibiting 6.56 to 21.32% of the phenotypic variation, and 16 QTLs for forage quality-related traits such as CP, crude fat, neutral detergent fiber (NDF), and ADF, explaining 5.79–41.72% of the phenotypic variation were identified (Asekova et al., 2016a,b; **Table 2**). Many of these QTLs showed high phenotypic variation and were expressed across different environments. Such QTLs with large phenotypic effects or the markers linked to these QTLs could be valuable in marker-assisted breeding programs. Alleles from PI 483463 contributed to the SFW QTL and most of the QTLs for forage quality traits, except CP content, indicating that wild soybeans like PI 483463 can serve as a valuable source of novel alleles for forage quality.

Forage traits are highly complex in nature and can be influenced by several other morphological and growth-related traits. Hence, although co-localization of QTLs may suggest pleiotropic effects of some genes and common regulation of the associated phenotypes, it is possible that genes directly related to forage quality may be different. The main-effect QTLs for forage quality traits and shoot fresh weight on chromosome 19 were co-localized (Asekova et al., 2016a,b), and exhibited high phenotypic variation for a majority of the traits. These QTLs spanned a genetic distance of 7 cM. Using a high-density bin map, several candidate genes for SFW were identified (Asekova et al., 2016b), one of which was *Dt1* (determinate stem), an ortholog of *Arabidopsis TERMINAL FLOWER1* (Liu et al., 2010). This gene was shown to be responsible for the determinate growth of soybean plants (Tian et al., 2010). Additional fine mapping and analysis of sequence variation (Asekova et al., 2016b) and tissue- and stage-specific expression of candidate genes from the QTLs showing co-localization need to be carried out to understand their contribution to explaining the phenotypic variation of the relevant trait.

Although few in number, the above-mentioned studies show the potential of soybean as an alternative forage crop. In particular, they show that wild soybeans carry novel alleles that can be introgressed into the desired cultivar for improved forage quality and yield. Furthermore, the markers tightly linked to the traits may assist in MAS and GS to improve productivity and quality of forage. Unfortunately, genomics-assisted breeding in soybean for forage traits is lagging far behind that for domestication-related traits, mainly due to the limited reports of studies involving genetic dissection of forage traits. It has been demonstrated that most of the rare alleles present in wild relatives of soybeans are absent in cultivated soybeans (Hyten et al., 2006). For traits such as fatty acid composition, it has been hypothesized that wild soybean may carry different sets of genes absent in cultivated soybeans (Pantalone et al., 1997; Kulkarni et al., 2017b), which, however, may be beneficial in improving a trait of interest. With the availability of genome sequences of both wild (*G. soja*; Li et al., 2014) and cultivated soybean (*G. max*; Schmutz et al., 2010), genetic mapping studies and identification of molecular variation within candidate genes controlling forage traits are expected to unravel functional usefulness of alleles from wild soybean relatives.

### Genetic Mapping of Morphological Traits in Cowpea

Cowpea exhibits significant variation in several morphological traits and yield, which are essential for its use as an animal feed. Although several cultivars of cowpea have been utilized extensively for forage, as monocrop or intercropped with cereals, few genetic studies have been conducted to investigate forage traits of the species. Cowpea is used generally as a dry grain and animal fodder crop. Its leaves are also a high-protein source. Using a RIL population Sanzi × Vita 7, Pottorff et al. (2012) identified a major QTL for leaf shape, *Hls* (hastate leaf shape), on the genetic map spanning a distance of 11 cM on linkage group 15. Further marker-trait association and analysis of synteny with *M. truncatula* and *G. max* identified an ortholog of the *EZA1/SWINGER* (AT4G02020.1) gene as the candidate gene for the *Hls* locus. Such genes may be utilized to improve the quality of cowpea leaves as a vegetable and forage, in addition to contributing to our understanding of the genetic control of leaf shape in legume crops. Delayed senescence, grain yield, and biomass yield under water stress significantly affect forage quality in cowpea cultivars. Muchero et al. (2013) genotyped a panel of 383 diverse cowpea accessions and a RIL population using an Illumina 1536 GoldenGate assay, and identified seven loci, five of which exhibited evidence of pleiotropic effects between delayed senescence, biomass, and grain yield.

Forage yield is associated with several other traits for which QTLs have been identified in cowpea; for instance, significant QTLs for maturity (Muchero et al., 2009b, 2011), flowering time (Andargie et al., 2013), and pod-length variation (Lo et al., 2017; Xu et al., 2017). Cowpea cultivars show extensive macrosynteny of about 85% with soybean (*G. max*) and 82% with *M. truncatula* (Muchero et al., 2009a), which will aid in comparative analysis to identify genomic regions related to forage quality and yield. With continued reduction in sequencing costs and advanced genotyping methods, linkage analysis and association mapping studies of cowpea are expected to increase and create a better understanding of the genetic architecture of traits important for forage breeding.

## Genomic Selection for Forage Traits in Legumes

Genetic variation is essential for understanding trait expression and for developing new genotypes (Kulkarni et al., 2013). The availability of a large set of SSR and SNP markers in a wide range of crops allows new prospects for crop improvement. These markers enable effective identification and characterization of genetic variation, tagging of QTLs useful for enhancing target traits, and manipulation of genetic variation in breeding populations (Xu and Crouch, 2008). Utilizing molecular markers to assist in the selection of desired traits in crop improvement programs has been an active area of research in the past few decades; however, results have been unsatisfactory for quantitative traits (Bernardo, 2008; Heslot et al., 2015). The MAS approach uses a small set of identified DNA markers linked to the QTL controlling a trait of interest. This, in essence, limits the expected success of MAS due to the small proportion of the genetic variation of the trait explained by each QTL. Genomic selection, on the other hand, offers simultaneous selection of hundreds of thousands of markers that densely span the entire genome to ensure that all genes are expected to be in LD with at least some of the markers (Bhat et al., 2016). It enables the use of all the associated marker information to develop a prediction model avoiding biased marker effects (Heffner et al., 2009) to estimate breeding values. Since GS captures small-effect QTLs (Desta and Ortiz, 2014) governing the majority of the phenotypic variation, including epistatic interaction effects (Deshmukh et al., 2014), it could be a highly useful strategy in trait improvement breeding programs.

There have been reports of successful combination of GBS-GWAS in identifying marker-trait associations for complex quantitative traits (Sonah et al., 2014), as envisioned by a large number of research articles in the last seven years (Zargar et al., 2015). These developments are expected to facilitate GS in a wide range of crop species, including forage legumes. Hayes et al. (2013) explored the potential and challenges of implementing GS successfully in forage crops. Several loci associated with forage traits have been identified in alfalfa using GBS-GWAS approaches as listed in **Table 2**. However, studies reporting GS for polygenic traits are lacking. Using GBS, Li et al. (2015) genotyped tetraploid alfalfa plants with 10,000 SNP markers and developed prediction equations using yield data from three locations. Their GS model for predicting total biomass yield, which was developed using a training population, had accuracies of up to 0.40 in the reference population. In a study by Annicchiarico et al. (2015), two genetically contrasting reference populations of alfalfa were genotyped by GBS and phenotyped in different environments for dry matter yield of half-sib progenies. They not only attained an accuracy of 0.35 using at least 10,000 SNP markers but also predicted a yield gain per unit time more than three times greater for GS compared to conventional selection for parent breeding value. Recently, Biazzi et al. (2017) performed GS of forage quality traits in alfalfa based on breeding values of parent plants. In this study, they used 11,450 polymorphic SNP markers to develop genome-enabled prediction to predict accuracy values for forage quality traits (**Table 2**). Leaf protein content and stem NDF digestibility displayed the best genome-enabled predictions, with accuracy values close to 0.40 and 0.30, respectively (Biazzi et al., 2017). These studies show that GS can accelerate genetic gain in alfalfa for forage yield (Li et al., 2015), dry matter yield (Annicchiarico et al., 2015), and quality traits (Biazzi et al., 2017). Although small in number, these studies can contribute better designs of GS strategies for alfalfa and other forage crops. In soybean, genome-enabled predictions with better accuracy values have been obtained for different traits such as soybean cyst nematode resistance (Bao et al., 2014), grain yield (Jarquin et al., 2014), and seed weight (Shu et al., 2013; Zhang et al., 2016). Several such empirical GS reports in different plant species indicate the feasibility of GS for yield and quality traits in forage legumes. With the increasing interest in soybean as an alternative forage legume, efforts to obtain genome-enabled predictions for forage quality and yield-related traits are expected to increase in the next few years.

At a time when the progress in molecular breeding has not benefited forage crops to the same extent as other major crop plants, effective implementation of GS has the potential to deliver better end results, mainly through facilitating multiple selection rounds within time periods conventionally used for single rounds (Hayes et al., 2013). This is possible only if accurate genomics-enabled breeding values are predicted for important traits. Several factors, including sample size and relatedness, marker density, gene effects, heritability and genetic architecture, and the extent of LD between markers and QTLs, are important in predicting the accuracy of GS models (Nakaya and Isobe, 2012; Desta and Ortiz, 2014). Since the application of GS does not eliminate phenotyping but replaces many of the selections associated with phenotyping based on whole-genome prediction, efficient high-throughput phenotyping methods for complex traits (Cabrera-Bosquet et al., 2012) could help to improve the accuracy of the prediction model (Desta and Ortiz, 2014). Although most of the key forage traits in legume crops are polygenic and inter-related, effective application of GS strategies offers an opportunity for breeders to enhance genetic gains.

## Improving Forage Digestibility by Genetic Manipulation

Forage crops play a significant role in agriculture and the animal food supply chain. Efforts to improve forage legumes for low or moderate heritability traits, such as digestibility, lignin content, nutritive value, and yield, through conventional phenotype-based selection approaches are inadequate due to cost and limits in logistics (Barrett et al., 2015). Rapid advancements in cellular and molecular biology and genetic transformation technology can provide unique methods to advance and supplement conventional breeding efforts. Recently, substantial improvement has been made in transgenic forage grasses and legumes. Protoplast and biolistic^TM^ methods have been widely used to generate transgenic plants in forage grasses, whereas the *Agrobacterium*-mediated transformation method has been applied for producing transgenic forage legumes (Spangenberg et al., 1997, 2001). Feed quality is a crucial factor for better health and performance of ruminants. Dairy firms in the United States are demanding high-quality forage even at the cost of yield (Martin, 2008). Therefore, improving forage digestibility is considered a prime goal of forage breeding programs aimed at providing high quality feeds to livestock. Fiber digestibility, protein quality, and reduction in CP degradation could be the target traits for genetic manipulation to improve nutritive value and enhance forage digestibility (Kumar, 2011).

Energy availability to animals fed with forages is restricted mainly due to low digestibility of plant cell wall material. Hence, major international research efforts in forage quality improvement have focused on improving access to enzymes involved in the biosynthesis of cell wall polysaccharides, cellulose, and hemicellulose. Lignin is one of the major chemical components of vascular plant cell walls and has a significant impact on forage quality. The concentration of lignin increases with plant maturity and has a negative correlation with forage digestibility. The lignin biosynthesis pathway and the genes involved in lignin biosynthesis are well known (Bonawitz and Chapple, 2010). Some of these genes have been targeted for genetic manipulation in dicotyledonous species such as alfalfa (Baucher et al., 1999; Shadle et al., 2007), *Arabidopsis* (Meyer et al., 1998), poplar (*Populus* sp.; Voelker et al., 2010), and tobacco (*Nicotiana* L. spp.; Sewalt et al., 1997). Recently, Verma and Dwivedi (2014) reviewed the genes encoding enzymes such as caffeic acid *O-*methyl transferase, cinnamyl alcohol dehydrogenase (CAD), 4-coumarate-CoA ligase, cinnamoyl-CoA reductase (CCR), ferulate-5-hydroxylase (F5H), phenylalanine ammonia-lyase, cinnamate-4-hydroxylase (C4H), 4-coumarate 3-hydroxylase (C3H), and peroxidases, which are targeted for genetic modification. Manipulating these genes in alfalfa, tobacco, and switchgrass (*Panicum virgatum* L.) improved forage digestibility in *in vitro* experiments (Getachew et al., 2011).

Several researchers have manipulated forage lignin content in alfalfa (**Table 3** and **Supplementary Table S1**). Reddy et al. (2005) developed transgenic lines of alfalfa with altered lignin content and composition by targeting three cytochrome P450 enzymes, C4H, coumaroyl shikimate 3-hydroxylase, and coniferaldehyde 5-hydroxylase (C5H), which catalyze monolignols, guaiacyl, and syringyl units, respectively. Downregulation of C4H and C5H reduced lignin content without a significant effect on lignin composition and resulted in increased digestibility. At the same time, downregulation of C4H, C3H, F5H, and hydroxycinnamoyl CoA:shikimate hydroxycinnamoyl transferase in alfalfa showed a negative impact on phenotypes such as impaired growth and biomass (Reddy et al., 2005; Shadle et al., 2007). There have been a few contradictory results related to lignin manipulation and digestibility. For instance, downregulation of *O-*methyl transferase (OMT) caused a 10-fold decrease in syringyl:guaiacyl content and increased *in vitro* digestibility in transgenic tobacco (Vailhe et al., 1996). In another study, transgenic alfalfa with downregulation of CAD did not affect Klason lignin content, but caused 50% reduction in the syringyl:guaiacyl ratio with increased *in vivo* digestibility (Baucher et al., 1999). Relationships between lignin content, composition, and *in vivo* digestibility improvement have been assessed by Guo et al. (2001a), with downregulation of OMT and caffeoyl-CoA *O-*methyl transferase in alfalfa transgenic plants found to exhibit complete syringyl lignin elimination with a significant increase in ruminant digestibility. Downregulation of C3H displayed a significant improvement in the digestibility of alfalfa forage. Such reports show that lignin content, rather than lignin composition, plays an important role in rumen digestibility. More recently, Jackson et al. (2008) demonstrated that *in vitro* digestibility improved in response to reduction in lignin content by downregulation of CCR and CAD in alfalfa. Cinnamoyl-CoA reductase and CAD function as branching points in the lignin biosynthesis pathway (targeting enzymes of the monolignol-specific branch), and are considered potentially suitable candidates for lignin modification.

**Table 3:** Genetic modification of lignin biosynthesis genes using antisense approach in alfalfa and its impact on forage digestibility.

Gene	Lignin content	Lignin composition	Digestibility	Reference
*Cinnamoyl CoA-reductase*	Decreased	S/G ratio increased	Increased	Jackson et al., 2008
*Cinnamyl alcohol dehydrogenase*	Unchanged	S/G ratio decrease	Increased	Baucher et al., 1999
*Cinnamyl alcohol dehydrogenase*	Decreased	S/G ratio decrease	Increased	Jackson et al., 2008
*Cinnamate 4-hydroxylase*	Decreased	S/G ratio decreased	Increased	Reddy et al., 2005
*Coumarate 3-hydroxylase*	Decreased	High H	Increased	Reddy et al., 2005
*Caffeate O-methyltransferase*	Decreased	S/G ratio decreased, 5-OH-G increased	Increased	Guo et al., 2001a,b
*Hydroxycinnamoyl-CoA: shikimate/guinate hydroxycinnamoyltransferase*	Decreased	High H	Increased	Shadle et al., 2007
*Caffeoyl-CoA O-methyltransferase*	Decreased	S/G ratio increased	Increased	Guo et al., 2001a,b
*Cinnamyl alcohol dehydrogenase*	Unchanged	S/G ratio decrease	Increased	Baucher et al., 1999
*Ferulate 5-hydroxylase*	Unchanged	S/G ratio decreased	Unchanged	Reddy et al., 2005
*Coumarate 3-hydroxylase*	Decreased	S/G ratio unchanged	_	Pu et al., 2009; Ralph et al., 2012
*O-methyltransferase*	Decreased	S/G ratio decreased	_	Marita et al., 2003
*Ferulate 5-hydroxylase/Coniferaldehyde 5-hydroxylase*	Decreased	S Reduced	_	Nakashima et al., 2008
*OMT × Caffeoyl-CoA O-methyltransferase*	Decreased	G decreased	_	Guo et al., 2001a
*Hydroxycinnamoyl-CoA: shikimate/guinate hydroxycinnamoyltransferase*	Decreased	S/G ratio decrease	_	Pu et al., 2009


*G, guaiacyl; H, p-hydroxyphenyl; 5OHG, 5-hydroxyguaiacyl; S, syringyl.*

Unlike alfalfa, cowpea and soybean have received little attention as forage crops, and hence no efforts have been made to manipulate genetically lignin and cell wall composition in these species. The limited genetic information (genes/QTLs identified) for forage traits in soybean and cowpea may be another reason. As mentioned earlier, Asekova et al. (2016b) identified several candidate genes from a significant QTL for SFW on chromosome 19, which was also found to co-localize with forage quality traits in soybean. Some of these genes encode enzymes influencing plant architecture, which may determine the quantity and quality of the forage produced. Recently, Li et al. (2017) isolated and characterized the dirigent domain in the *GmDIR22* gene in response to abiotic and biotic stresses. The principal roles of the dirigent gene family are in defense responses and fiber biosynthesis (Davin and Lewis, 2003; Berim et al., 2008). In addition, these genes play an important role in plant secondary metabolism, including lignan and lignin formation (Dalisay et al., 2015; Effenberger et al., 2015). Hence, genes from this family may also be important candidates for genetic manipulation to improve lignin content in forage soybean. Similarly, the development of the GENOSOJA database for *in silico* predictions on soybean transcriptomes related to enzymes and proteins involved in cell wall, lignin, and fatty acid metabolism is expected to provide valuable information on the regulation of coding gene expression (Nascimento et al., 2012). Moreover, Pestana-Calsa et al. (2012) identified *in silico* 49 reads associated with cell wall components (polysaccharides and lignin) in soybean. Such candidates can also be targets for genetic manipulation to improve forage digestibility. In addition, genes identified for lignin biosynthesis in alfalfa and other crop species can be exploited for genetic manipulation using soybean or cowpea as a heterologous system.

## Achieving Adequate Forage Yields Through Integrative Approaches

**FIGURE 1:**
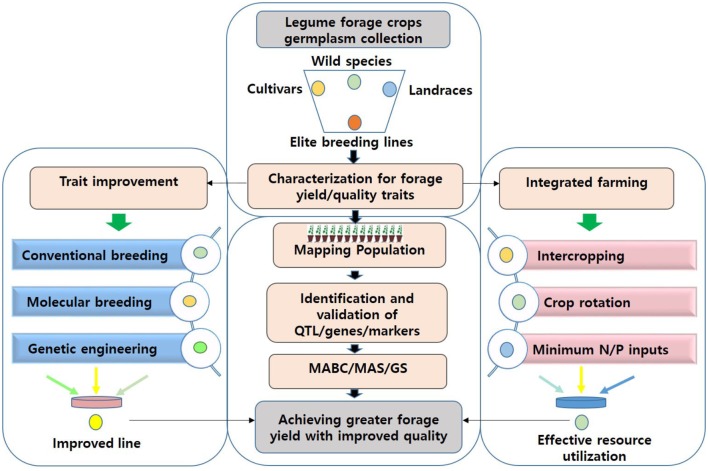
An integrated approach for achieving sufficient fodder and feed for livestock. Several cultivated or wild relatives of legume crops are known to inherently possess high-quality forage characteristics. Such germplasm can be scrutinized to identify QTL/gene/SNP associated with forage quality parameters or yield-related traits. This information can either be utilized in MAS/GS for genomics-assisted breeding or in genetic engineering for improving forage digestibility and associated traits in current forage legume cultivars. Implementation of sustainable crop management practices such as intercropping may help to achieve high-quality forage with greater yields, and assure food security in a future scenario of climate variability. MABB, marker-assisted backcross breeding; MAS, marker-assisted selection; QTL, quantitative trait loci; SNP, single nucleotide polymorphism; GS, genomic selection; N, nitrogen; P, phosphorous.

Due to the significant progress made in access to food in many countries, demand for animal protein sources such as milk and meat is ever-increasing. Because the overuse of grasslands is becoming a serious problem, achieving quality feed in sufficient quantities will be challenging. Forage legume crops are of immense significance in providing quality fodder and feed to livestock, the major source of protein food for humans. Forage yield is associated with several other traits, such as plant height, maturity, pod length, number of pods, and leaf and stem morphology. Thus, in order to achieve high forage yield, we emphasize the importance of applying an integrated approach, involving trait selection and evaluation, genetic improvement and manipulation, and integrated farming (**Figure 1**). A multi-site evaluation of available germplasm and other genetic resources, through improved, cost-effective, and high throughput phenotyping technologies, needs to be conducted to assess trait performance. Materials of high value can be utilized as genetic resources for improving the target traits, first by identifying the markers/QTLs/genes controlling desired traits and using them in selection procedures (through MAS/GS). In addition, improving forage digestibility through genetic engineering can enhance animal performance. Recent advancements in genetic and genomic technologies have facilitated genetic dissection of complex traits. Further genomic research is expected to reveal genes, and associated molecular markers, controlling forage quality and yield-related traits in key legume crops. The advent of high-throughput genotyping platforms further enables screening large numbers of plants in a short period to unravel the genetic makeup of the plants. When integrated, these approaches are expected to facilitate the genomics-assisted improvement of forage legume cultivars.

Improved cultivars alone may not be sufficient for providing adequate quantities of quality forage unless they are exploited further in effective crop management practices. Intercropping forage legumes with cereal crops offers the potential for improving forage quality and yield, consequently increasing livestock production. Intercropping is principally beneficial due to dissimilarities in ecological characteristics and growth of the intercropped varieties. This results in optimization of the intercropped species to improve yield and economic income per unit area (Sun et al., 2014). Growth habits and growth rates of component crops, planting density, differences in root architecture, and maturity are some of the factors that influence competition for water and mineral nutrients among the component crops in intercropping systems (Ghosh et al., 2009). In addition, legume-grain intercropping intensifies land use and improves yield and soil fertility, limits weed growth, and reduces the risk of crop failure (Rusinamhodzi et al., 2012). Therefore, it has gained much significance for the development of sustainable food production systems, predominantly in cropping systems with inadequate external inputs (Adesogan et al., 2002). Crop management designs, specific for each kind of intercropping system and geographic region, which facilitate interspecific root interactions, minimize competition for resources, require minimum N and P input, and allow maximum radiation absorption need to be identified and put into practice to enhance forage yield. Achieving greater yields through the implementation of sustainable agricultural practices, in addition to continued technological advancements, will play a crucial role in mitigating the effects of climate change to ensure food security.

## Summary and Future Perspectives

Legumes are grown across the world as forage and fodder for animals. However, recent climate changes adversely influencing forage quality traits have become a serious concern for farmers and cattle growers. This alarming situation necessitates the development of forage legume cultivars that can withstand such climatic fluctuations while retaining all the inherent quality traits and nutritive value. In this review, we assessed the potential of three important forage legumes, alfalfa, soybean, and cowpea, for the prospect of providing quality forage and feed with significant yields. Alfalfa has long been cultivated and used as forage and feed for cattle and horses across the world. On the other hand, soybean and cowpea, important food crops for humans, are used as grain fodder for cattle. The information presented in this review show that all the three legume crops have the potential for providing forage as well as fodder and feed for livestock. Significant progress in the field of molecular genetics and genomics offers the means for genetic improvement of quality and yield traits in forage legume crops. The availability of large-scale genomic resources for alfalfa, as well as soybean and cowpea, has helped to identify a number of molecular markers. These markers can be utilized to identify QTLs linked to forage quality and yield traits. In alfalfa, many researchers have employed linkage or association mapping approaches to identify markers/QTLs for forage biomass productivity under drought conditions. Information relating to these markers can be used to develop drought-tolerant cultivars. Similarly, forage quality traits, primarily protein concentrations, and cellulose and lignin content can be improved using marker-assisted breeding approaches. Another approach for employing marker-trait associations in crop improvement is through GS, which allows simultaneous use of markers spread across the chromosomes for enhancing trait performance.

Increasing biomass density while retaining forage quality may be challenging, owing to concurrent increases in lignin content in plant cell walls. Hence, researchers have dedicated efforts in manipulating lignin composition genetically by targeting genes in the lignin biosynthesis pathway in alfalfa plants without drastically affecting other traits. Similar efforts made in other crops provide information about target genes, which can be employed in genetic manipulation using soybean or cowpea as a heterologous system. The results appear to be promising and indicate a crucial role for efficient application of transgenic technology in improving forage digestibility; however, use of transgenic plants in the field is regulated, and require additional permissions. The application of advanced technology such as CRISPR/cas9 may circumvent these permissions and provide significant benefits in terms of forage quality as well as related trait improvements. The ongoing research progress is slow in second-generation traits (nutrition, quality, aroma, flavor, bioenergy, etc.) and products with environmental or other benefits contributing to sustainable food chains, and needs to be accelerated. Forage legume crops with genetically improved quality traits may be beneficial, both for digestibility of animal feed and for the environment.

## Author Contributions

KPK, RT, and SA wrote the manuscript. KPK conceptualized the overall structure, and edited the manuscript. KPK, RT, and SA prepared illustrations, figures, tables, and references. JTS and JGS contributed critical comments to the draft. J-DL conceptualized, critically edited, and approved the manuscript. All the authors reviewed the draft.

## Conflict of Interest Statement

The authors declare that the research was conducted in the absence of any commercial or financial relationships that could be construed as a potential conflict of interest.
