# Supplementary material for: Harnessing the Potential of Forage Legumes, Alfalfa, Soybean, and Cowpea for Sustainable Agriculture and Global Food Security
Source: Front Plant Sci. 2018 Sep 19;9:1314. doi: 10.3389/fpls.2018.01314 (PMC6157451; doi:10.3389/fpls.2018.01314)
Supplement: Supplementary file 1 [file Table_1.pdf]

Table S1. **Genetic modification of lignin biosynthesis genes in different plant species**

Species	Gene	Modification approach	Lignin content	Lignin composition	Reference
Arabidopsis	*F5H/CALD5H*	Over-expression fusion construct	Increased	S units increased	Meyer et al., 1998. Proc. Natl. Acad. Sci. U.S.A. 95, 6619-6623.
	HCT	Sense/antisense RNAi	Decreased	H units increased, S units decreased	Hoffmann et al., 2004. Plant Cell 16, 1446-65.
	4CL	Antisense	Decreased	G units decreased	Lee et al., 1995. Plant Mol. Biol. 28, 871–884.
	OMT C4H: F5H1	Over-expression	Increased	S units increased	Vanholme et al., 2010. Plant J. 64, 885–897.
	CCR	Antisense	Decreased	S/G ratio decreased	Goujon et al., 2003. Planta 217, 218–228.
Aspen	*F5H/CALD5H*	Sense /antisense 4CL and sense CAld5H genes	Unchanged	S/G ratio increased	Li et al., 2003. J. Plant Res. 116, 175–182.
	4CL	Antisense	Decreased	S/G ratio unchanged	Hu et al., 1999. Nat. Biotechnol. 17, 808–812.<br>Li et al., 2003. J. Plant Res. 116, 175–182.
	4CL × CALD5H	Antisense and sense	Decreased	S/G ratio increased	Li et al., 2003. J. Plant Res. 116, 175–182.
Birch (*Betula platyphylla* × *Betula pendula*)	*CCR*	Overexpression/ suppression	Increased/ Decreased	NA	Zhang et al., 2015. Physiol. Plant. 154, 283-296.
Leucaena leucocephala	*OMT*	Antisense	Decreased	S units decreased, cellulose increased	Rastogi and Dwivedi 2006., Biotechnol. Prog. 22, 609–616.
Nicotiana benthamiana	*HCT*	RNAi	Decreased	S units decreased	Hoffmann et al., 2004. Plant Cell 16, 1446-65.
Nicotiana tabaccum transformed	*CCR*	Antisense	Decreased	S/G ratio increased	Srilakshmi et al., 2011. Plant Cell Rep. 30, 2215–2231.
	CCR	Sense up-regulation	Increased	S units increased, G units decreased	Srilakshmi et al., 2011. Plant Cell Rep. 30, 2215–2231.
Norway spruce (*Picea abies* L.) Karst	*CCR*	Antisense	Decreased	H/G ratio decreased	Wadenback et al., 2008. Transgenic Res. 17, 379–392.
Poplar	*CCR*	Antisense	Decreased	S/G ratio decreased	Leple et al., 2007. Plant Cell 19, 3669-3691.
	CAD	Antisense	Decreased	S units increased	Lapierre et al., 1999. Plant Physiol. 119, 153–163.
Switchgrass	*COMT*	RNAi	Decreased	S/G ratio decreased	Baxter et al., 2016. Euphytica 209, 341.
	MTHFR/COMT	RNAi/antisense	Decreased	S/G ratio decreased	Liu et al., 2017. Front. Plant Sci. 8, 982.
Tall fescue	*CAD fl*	Sense or antisense	Decreased	S/G ratio decreased	Chen et al., 2003. Plant Biotechnol. J. 1, 437–449.
Tobacco (*Nicotiana* L. spp.)	*C4H*	Antisense	Decreased	S/G ratio decreased	Blee et al., 2001. Phytochemistry 57, 1159–1166.<br>Sewalt et al., 1997. J. Agric. Food Chem. 45, 1977–1983.
	C4H	Sense suppression	Decreased	S/G ratio decreased	Sewalt et al., 1997. J. Agric. Food Chem. 45, 1977–1983.<br>Sewalt et al., 1997. Plant Physiol. 115, 41–50.
	C4H	Over-expression	Unchanged	S/G ratio unchanged	Sewalt et al., 1997. J. Agric. Food Chem. 45, 1977–1983.<br>Sewalt et al., 1997. Plant Physiol. 115, 41–50.
	PAL	Sense suppression	Decreased	S/G ratio decreased	Franke et al., 2000. Plant J. 22, 223–234
	OMT	Antisense	Decreased	S/G ratio decreased	Dwivedi et al., 1994. Plant Mol. Biol. 26, 61–71.

Species	Gene	Modification approach	Lignin content	Lignin composition	Reference
Tobacco (*Nicotiana* L. spp.)	*OMT*	Antisense	Decreased	Unchanged	Ni et al., 1994. Transgenic Res. 3, 120-126.<br>Ogras et al., 2000. Turk. J. Bot. 24, 221–226.
	OMT	Antisense	Unchanged	S/G ratio decreased, new 5-OH-G accumulated	Atanassova et al., 1995. Plant J.8, 465–477.
	OMT	Sense	Unchanged	Unchanged	Atanassova et al., 1995. Plant J. 8, 465–477.
	F5H/CALD5H	Over-expression	Decreased	S units increased	Franke et al., 2000. Plant J. 22, 223–234.
	4CL	Sense	Decreased	S units decreased	Kajita et al., 1996. Plant Cell Physiol. 37, 957–965.
	4CL	Antisense	Decreased	S units decreased	Kajita et al., 1996. Plant Cell Physiol. 37, 957–965.
	4CL	Antisense	Unchanged	S units decreased	Kajita et al., 1997. Plant Physiol. 114, 871–879.
	CCR	Antisense	Decreased	S/G ratio increased	Piquemal et al., 1998. Plant J.13, 71–83.
	CCR	Antisense	Decreased	G units decreased	Ralph et al., 1998. Proc. Natl. Acad. Sci. U.S.A. 95, 12803–12808.
	CCR	Partial sense suppression	Decreased	S/G ratio increased	O'Connell et al., 2002. Transgenic Res. 11, 495–503.
	CAD	Antisense	Unchanged	S/G ratio decreased	Halpin et al., 1994. Plant J.6, 339–350.
	CAD	Antisense	Unchanged	S/G ratio increased	Higuchi et al., 1994. J. Biotechnol. 37, 151–158.
	CAD	Antisense	Unchanged	More aldehydes	Hibino et al., 1995. Biosci., Biotechnol. Biochem. 59, 929–931.<br>Higuchi et al., 1994. J. Biotechnol. 37, 151–158.
	CAD	Antisense	Unchanged	S/G ratio decreased, more cinnamaldehyde	Yahiaoui et al., 1998. Planta 204, 8–15.
	CAD	Antisense	Unchanged	G and S units decreased, more aldehydes	Baucher et al., 1996. Plant Physiol. 112, 1479-1480.
	CAD	Antisense	Unchanged	S units decreased	Pinchon et al., 2001. Plant Physiol. 126, 145–155.<br>Pinchon et al., 2001. Phytochemistry 57, 1167–1176.<br>Vailhe et al., 1998. J. Sci. Food Agric.76, 505–514.
	CAD	Antisense	Decreased	S/G ratio increased	O'Connell et al., 2002. Transgenic Res. 11, 495–503.
	CCR × *CAD*	Antisense; cross-pollinated	Decreased	S/G ratio decreased	Chabannes et al., 2001. Plant J. 28, 271–282.<br>Chabannes et al., 2001. Plant J. 28, 257–270.
	OMT × *CCoAOMT*	Antisense	Decreased	S/G ratio decreased	Zhong et al., 1998. The Plant Cell 10, 2033-2046.
	OMT × *CCoAOMT*	Antisense	Decreased	S units decreased	Huayan et al., 2002. Chin. Sci. Bull. 47, 1092-1095.
	OMT × *CCR*	Antisense; cross-pollinated	Decreased	S units decreased	Pinchon et al., 2001. Phytochemistry 57, 1167–1176.
	OMT × *CCoAOMT*	Antisense	Decreased	S units decreased	Pinchon et al., 2001a. Plant Physiol. 126, 145–155.
	OMT × *CAD*	Antisense	Unchanged	Unchagned	Abbott et al., 2002. Plant Physiol. 128, 844–853.
	OMT × *CAD*	Sense; single chimeric transgene	Decreased	S units decreased	Abbott et al., 2002. Plant Physiol. 128, 844–853.

Species	Gene	Modification approach	Lignin content	Lignin composition	Reference
Tobacco (*Nicotiana* L. spp.)	*OMT* × *CCR* × *CAD*	Sense	Decreased	S units decreased	Abbott et al., 2002. Plant Physiol. 128, 844–853.
	PAL	Sense	Decreased	Unchagned	Elkind et al., 1990. Proc. Natl. Acad. Sci. U.S.A. 87, 9057–9061.<br>Bate et al., 1994. Proc. Natl. Acad. Sci. U.S.A. 91, 7608–7612.
	PAL	Sense	Increased	Unchagned	Howles et al., 1996. Plant Physiol. 112, 1617–1624.
	CAD	Antisense	Unchanged	NA	Vailhe et al., 1998. J. Sci. Food Agric.76, 505–514.
	PAL	Antisense	Decreased	S/G ratio increased	Elkind et al., 1990. Proc. Natl. Acad. Sci. U.S.A. 87, 9057–9061.<br>Sewalt et al., 1997. J. Agric. Food Chem. 45, 1977–1983.<br>Sewalt et al., 1997 Plant Physiol. 115, 41–50.
	COMT	Sense or antisense	Unchanged	S/G ratio decreased	Vailhe et al., 1996. J. Sci. Food Agric. 72, 385–392.
	COMT	Antisense	Decreased	S/G ratio decreased	Sewalt et al., 1997. J. Agric. Food Chem. 45, 1977–1983.<br>Sewalt et al., 1997. Plant Physiol. 115, 41–50.

Antisense, transgenic plants with an antisense construct; G, guaiacyl; H, p-hydroxyphenyl; 5OHG, 5-hydroxyguaiacyl; S, syringyl; Sense, transgenic plants with a sense construct.

4CL, 4-coumarate:coenzyme A ligase; C3H, p-coumarate 3-hydroxylase; C4H, cinnamate 4-hydroxylase; CAD, cinnamyl alcohol dehydrogenase; CALD5H, Coniferaldehyde 5-hydroxylase; CCoAOMT, caffeoyl-CoA O-methyltransferase; CCR, cinnamoyl CoA-reductase; COMT, caffeate O-methyltransferase; F5H, Ferulate 5-hydroxylase; HCT, hydroxycinnamoyl-CoA:shikimate/guinate hydroxycinnamoyltransferase; MTHFR, Methylenetetrahydrofolate reductase; OMT, O-methyltransferase; PAL , phenylalanine ammonia-lyase.
